# UniVRse: protocol for a pilot randomised controlled trial of virtual reality cognitive-behaviour therapy for students with social anxiety

**DOI:** 10.1186/s40814-026-01771-4

**Published:** 2026-02-05

**Authors:** Cassie M. Hazell, Josie Malinowski, Bethany Edwards, Aislinn D. Gómez Bergin, Clio Berry, Maria Flynn, Nina Smyth, Jo Birkett

**Affiliations:** 1 Department of Psychological Interventions, School of Psychology, University of Surrey, Guildford, GU2 7XH UK; 2 School of Social Sciences, University of Westminster, London, W1W 6UW UK; 3 School of Psychology, University of East London, London, E15 4LZ UK; 4 Responsible Ai UK, School of Computer Science, University of Nottingham, Innovation Park, Triumph Road, Nottingham, NG7 2TU UK; 5 Department of Primary Care and Public Health, Brighton and Sussex Medical School, Falmer, BN1 9PH UK; 6 Institute of Psychiatry, Psychology, & Neuroscience, King’s College London, 16 De Crespigny Park, SE5 8AF London, UK

**Keywords:** Virtual reality, Social anxiety, Student mental health, Pilot RCT

## Abstract

**Background:**

Social anxiety is prevalent amongst university students. Cognitive-behaviour therapy (CBT), and graded exposure techniques in particular, is an effective intervention for social anxiety. However, there are a number of barriers preventing the delivery of CBT to students who are socially anxious. Delivering this intervention using virtual reality (VR) can address these implementation issues. We have co-developed with a group of students a VR-CBT intervention (UniVRse) specifically for members of this student group with social anxiety.

**Methods/design:**

The present study is a pilot randomised controlled trial conducted in the United Kingdom of the UniVRse intervention compared to a wait-list control group. The aim of the trial is to determine whether a definitive trial is justified by assessing study recruitment, retention, and acceptability, as well as establishing the effect size on the co-primary outcomes for the definitive trial sample size calculation. We aim to recruit 90 socially anxious students—45 in each trial arm. The trial will adopt a mixed-methods approach. We will collect quantitative data at baseline (T0) and post-intervention 6 weeks later (T1). We will invite participants randomised to the intervention arm to complete a qualitative exit interview.

**Discussion:**

The results of this pilot trial will be used to determine whether a definitive trial is justified, and to inform the refinement of the UniVRse programme and trial procedures. In the longer term, the UniVRse intervention has the potential to be an effective and accessible psychological intervention for students with social anxiety.

**Trial registration:**

Clinicaltrials.gov, NCT05704868. Registered 30th January 2023

## Background

Concern for the psychological wellbeing of university students has grown over recent years [1], driven by several studies evidencing the high prevalence of depression and anxiety in this group [2, 3]. By comparison, students’ anxiety in the context of social situations (i.e. social anxiety) has been investigated to a lesser extent but is equally commonplace [4]. The available evidence suggests that 10% of UK university students meet criteria for severe social anxiety [5], with high rates evident elsewhere in the world as well [6–8].

Social anxiety in any population can be a life-limiting condition due to its psychological and physiological impacts, including loneliness, isolation, and relationship difficulties [9–11]. Amongst students, there are additional sequelae that arise because of the pressure to be ‘sociable’ when at university [12, 13]. Being socially anxious is associated with reduced wellbeing [14] and self-esteem [15], and increased risk of alcohol-related problems [16] amongst students. There is also evidence that being socially anxious is associated with alterations in the hypothalamic–pituitary–adrenal (HPA) axis, which is the major stress response system [17]. Social anxiety can also undermine academic functioning [8], including hindering learning [18] and reducing communication with teaching staff [19]; ultimately resulting in poorer educational attainment [20] and impeding the student experience [19]. Social anxiety may be global, affecting students in all social situations, or context-specific, presenting only when students are in the university environment [21].

The cognitive-behavioural model of social anxiety by Heimberg and colleagues [22] posits that social anxiety is driven by the belief that others are judging and making negative evaluations of you, and that this belief is maintained by attending to both internal cues (e.g. feeling hot and shaky) and vigilance of external stimuli (e.g. facial expressions of others) [23]. The Heimberg model [22] provides the framework for one approach to cognitive-behaviour therapy (CBT) for social anxiety, specifically the use of psychoeducation and graded exposure techniques as recommended by National Institute of Health and Care Excellence (NICE) guidelines [24].

Exposure-based techniques are an essential component of effective CBT for social anxiety [25], and have been demonstrated to be equally as effective in improving social anxiety as other cognitive-behavioural techniques such as cognitive restructuring [26]. Progression through exposure hierarchies is directly associated with reductions in social anxiety symptoms [27]. There is also some evidence of synchronous changes in cortisol levels following CBT [28] or exposure therapy [29] for anxiety. However, despite the effectiveness of exposure for social anxiety, there are several barriers preventing its implementation amongst the student population. Real-world exposure work is problematic in that it compromises confidentiality and does not afford the therapist any control over the social environment. For example, a therapist is commonly present or at least nearby during the *in-vivo* exposure, which may risk disclosure that the student has mental health difficulties and/or is engaged in therapy. Moreover, university environments are changeable, meaning the therapist cannot guarantee that the anxiety triggers they plan to work on will be present. Virtual reality (VR) offers an alternative mode of delivering exposure-based CBT that addresses these barriers.

VR-CBT for social anxiety applies graded exposure techniques to virtual simulations of the feared social environments. Patients can engage in VR-CBT in private spaces, therein protecting their privacy, and the programming of the virtual environment gives control over the presence and nature of anxiety-related triggers in line with grading procedures. An additional benefit of VR is that patients are more willing to engage in the exposure work as they know it is not real, yet they nonetheless mentally and physically respond as if it is [30]. Meta-analyses have evidenced large treatment effects of VR exposure for social anxiety disorder in the general population [31–33]. The treatment effects are equivalent to real-world exposure [32], and, in some studies, have even been found to be superior, with longer-lasting effects [34]. To further support the scalable implementation of VR-CBT, newer programmes are moving towards being self-guided, removing the need for therapist input. Again, these self-guided versions of VR exposure produce equivalent large treatment effects [35].

Using VR to deliver CBT for social anxiety, therefore, holds great promise as a means of providing an effective and accessible intervention for students with social anxiety. To our knowledge, there is only one small study that has piloted a VR–CBT intervention specifically for students with social anxiety. The authors found that virtual exposure to a public speaking scenario reduced public-speaking anxiety and physiological anxiety symptoms [36]. This study was a promising first step in using VR to support students with social anxiety, yet more work is needed to further evidence the utility of this approach and expand the focus of the intervention beyond public speaking encounters. There is a need to develop an intervention that utilises evidence-based therapeutic models and cutting-edge technology to target context-specific social anxiety amongst vulnerable students.

### Aims

The long-term aim of this programme of work is to test whether the UniVRse intervention is effective at reducing both global and context-specific social anxiety amongst students. Before we can address this aim, we must first conduct a pilot randomised controlled trial with the following aims:

1. To determine whether a definitive trial is justified by assessing study recruitment, retention, and acceptability;
2. To establish the effect size on the co-primary outcomes for a sample size calculation for a definitive trial.

A definitive trial will be justified if we are able to successfully recruit and retain participants in the trial, and if both the intervention and research protocols are deemed acceptable to participants. We will operationalise this as collecting complete data on one of the co-primary outcomes for at least 80% of the final sample, and for attrition from the study (i.e. meaning no data are provided at Time 1) to not exceed 40%. We will also use the responses on the participant experience measures and exit interviews to further understand acceptability.

Although the primary aim of the pilot trial is to address feasibility questions, it is also important to determine whether there is a signal of efficacy in favour of the trial intervention to further justify the need for a definitive trial [37]. Therefore, we will test for a signal of efficacy in favour of the UniVRse programme over the control condition; operationalised as the between-group effect sizes on the co-primary outcomes favouring UniVRse.

## Methods

### Design

**Fig. 1:**
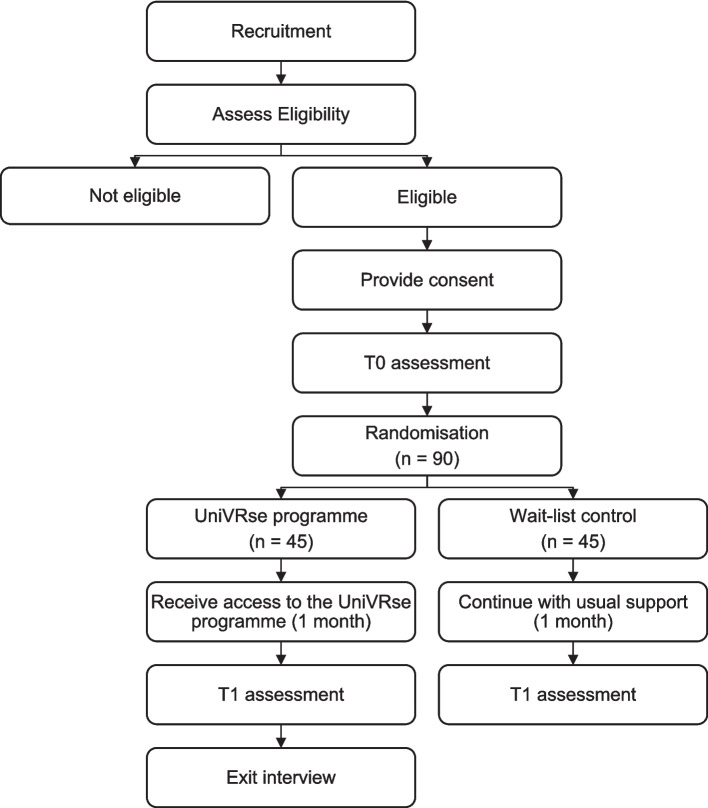
UniVRse trial CONSORT diagram

This study is a single-blind external pilot randomised controlled trial with two parallel arms: (1) UniVRse and (2) wait-list control; with optional exit interviews. See Fig. 1 for CONSORT diagram, and see Table 1 for SPIRIT checklist.

**Table 1:** SPIRIT checklist

	Study period					
	Enrolment	Allocation	Post-allocation			
Timepoint	*-t*_*1*_	0	*T0*	*Intervention*	*T1*	*T2*
Enrolment:						
Eligibility screen	X					
Informed consent	X					
Random allocation		X				
INTERVENTIONS:						
UniVRse programme				X		
Wait list control				X		
ASSESSMENTS:						
T0 assessment			X			
T1 assessment					X	
Exit interviews						X

### Randomisation and blinding

We will randomise participants to one of the trial arms after they have completed the baseline (T0) assessment. Randomisation will be at a 1:1 group allocation ratio using a permuted blocks algorithm with varying block sizes. The research team will be blinded to the size of the blocks and will have no influence over the outcome of the randomisation. The randomisation sequence will be set up and tested by an independent statistician using Sealed Envelope. The Principal Investigator (PI) will receive notification of each participant’s arm allocation via emails from Sealed Envelope once the T0 assessment has been completed.

Where participants elect to complete the assessments with the support of a research team member, we will ensure that this research team member will be blind to participants’ group allocation. Participants may elect to complete follow-up (T1) assessments independently, meaning they will not be subject to any biases associated with unblinding of the research team. Where T1 assessments are conducted with researcher support, this member of the research team will be blinded to the outcome of the randomisation. If a researcher becomes unblinded before the T1 assessment, a different member of the research team will be allocated to complete the assessment. If blinding is broken during an assessment, then the researcher will cease data collection and contact the PI or offer the participant the opportunity to complete the assessments independently. A different blinded member of the research team will then be allocated to finish the assessment. Any incidents of unblinding will be recorded and reported in any publications.

### Participants

Participants will be recruited from a single university in London. The study promotional materials will be displayed around campus and shared via student mailing lists and university social media outlets. The materials will include the project website and email address so that students can express an interest in participating in the study directly. We plan to recruit 90 participants (45 per arm). The recruitment target is based on guidance by Whitehead et al. [38] that 25 participants per arm are needed for a pilot trial prior to a main trial to detect a small–medium effect size, while allowing for an attrition rate of up to 40%. We will ask participants to complete a demographics questionnaire so that we can describe our sample.

We will assess whether participants are eligible for inclusion in the trial via a self-report survey. For participants to be eligible to participate in the trial, they must be (1) a current student at the trial site; (2) able to read and write independently in English; (3) identifying as wanting help to feel more confident when at university; and (4) experiencing social anxiety that is context-specific to the university environment. There are no specific diagnostic criteria that correspond to the mental health need we are targeting. Instead, we will operationalise this context-specific social anxiety as a score of ‘moderate’ or ‘often’ on at least one of four items from the Hazell University-Specific Social Anxiety Scale (HUSSAS) (items 5, 9, 12, and 17). These four items reflect each of the scenarios included in the UniVRse intervention (see Section 7.4).

We will exclude any persons who (1) have any conditions that prevent them from using VR, including (a) photosensitive epilepsy, (b) a visual impairment that cannot be corrected with glasses/lenses, (c) a balance disorder, or (d) a significant auditory impairment; (2) have current and active plans to end their life; or (3) have confirmed plans to receive a psychological therapy within the next 6 weeks. We define a psychological therapy as any intervention guided by an evidence-based model that targets a mental health-related outcome.

We have further exclusion criteria in place for the saliva sample collection as we cannot accurately measure cortisol in those who are (1) pregnant; (2) currently breastfeeding; (3) currently taking any medications; (4) having any serious medical condition; or (5) believing they currently have COVID-19. Participants may still take part in the main trial if they are ineligible for the cortisol sub-study.

### UniVRse

The UniVRse programme has been co-produced with students with lived experience of social anxiety and/or feeling unconfident at university (see “Public and patient involvement” section for more information). The UniVRse programme is a Virtual Reality (VR) cognitive-behavioural therapy (CBT) intervention based on the Heimberg model [22] of social anxiety, using graded exposure techniques to reduce social anxiety amongst students. The model posits that persons attend to both internal cues and external events, leading to the development and maintenance of beliefs that others are judging and making negative evaluations of them. These beliefs lead to behavioural responses including escape and avoidance, which consequently maintain the beliefs. Graded exposure is a core technique used to challenge unhelpful cognitions through habituation to the feared situation, and to provide the opportunity to practice previously avoided behavioural skills [24].

Within this intervention, participants are ‘gradually exposed’ to the feared situation, preventing avoidance and challenging any negative beliefs that are maintaining the anxiety. To achieve this, participants are able to enter virtual simulations of university-based situations that cause them anxiety and practice being in those scenarios. Participants start with a version of the scenario that has fewer anxiety-related triggers, and graduate to levels with more of these triggers. The UniVRse programme is a self-help intervention, meaning that it does not involve or require support from a therapist. Instead, participants are guided through the UniVRse programme by a virtual mentor called ‘Sam’. Sam provides psychoeducation, instructions, support, and encouragement.

Participants will have a degree of flexibility with regard to the session structure of the UniVRse programme, with some fixed parameters. Each session should not exceed 30 min, as being in VR beyond this can cause headaches and eyestrain [39]. Participants will receive a prompt within the virtual environment when they have been using the programme for 30 min. We will give participants access to the VR kit for a month post-randomisation—during this time we recommend that participants complete at least six 30-min sessions. Completing six sessions will constitute completion of the intervention—this is the minimum number of sessions required to complete the introductory psychoeducation, all of the levels in all of the scenarios, and the debrief. We will consider participants to have been ‘exposed’ to the intervention after at least two 30-min sessions. To accommodate the varied schedules of our student participants, they have full control over when they complete these sessions, including the spacing and timing of sessions.

At the start of the UniVRse intervention, participants are presented with a ‘tutorial’ in which Sam gives instruction on how to interact with the virtual environment and provides relevant psychoeducation. Participants are given information on social anxiety, ‘fight or flight’, avoidance as a means of maintaining anxiety, and anxiety habituation. After the tutorial has been completed, participants can select one of four university-based scenarios that they would like to practice being in. The scenarios are: (1) attending a lecture, (2) delivering a presentation, (3) contributing to an online seminar, and (4) group work in a seminar. Within each scenario, there are five graded levels, from the least to most anxiety-inducing. The levels are graded in terms of the number and magnitude of anxiety-related triggers. For example, when in the ‘delivering a presentation’ scenario, the lower levels have a smaller and friendlier-seeming audience, whereas the higher levels have a larger audience that appears more disinterested and includes avatars of teaching staff, with the participant additionally being required to answer a question for which they have not been able to prepare an answer.

#### Hardware

The UniVRse intervention has been designed to be used using the Meta Quest 2. This VR kit is self-contained, meaning it does not require a connection to a computer to work, and is designed for domestic use. Participants will be given the choice of either coming onto campus at the trial site to use the VR kit, or to take it away and use it at home. Guidance on how to use the VR kit is given within the virtual environment, and we have produced a set of bespoke instructions alongside this.

#### Personalisation

With the goal of increasing engagement, there are aspects of the UniVRse intervention that participants can personalise to fit their needs. At the start of the programme, participants can choose the gender and ethnicity of the intervention guide ‘Sam’. Participants also have choice over which scenarios they enter and in what order. Participants are advised to challenge their own avoidance behaviours and choose the scenarios that make them feel most anxious. They can repeat levels and scenarios or choose to move on depending on whether they feel their anxiety has habituated. The higher levels in a particular scenario will not be unlocked, however, until the preceding levels have been completed to ensure that the graded exposure remains hierarchical.

### Control condition

Participants allocated to the control condition will join a waitlist for the UniVRse intervention. Control participants will be offered the UniVRse intervention once the trial has been completed. These participants will be able to continue with any health treatment/support they are currently receiving. This treatment or support may include active monitoring, meetings with academic advisors or support workers, or psychiatric medication. Participants in the control condition may commence psychological therapy during the trial, but they must not have plans to do this at the time of providing consent (as per exclusion criteria). We will not be controlling for any aspects of standard treatment/support in the analysis.

### Measures

We are piloting two co-primary outcome measures: (1) the Social Phobia Inventory (SPIN) [40]; and (2) the Hazell University-Specific Social Anxiety Scale (HUSSAS). The SPIN has 17 items assessing general social anxiety. The validation of the original five-factor solution of the SPIN has been inconsistent [41]. More recent research instead supports a three-factor model [42], with all subscales demonstrating strong internal consistency (Cronbach’s *α*s ≥ 0.77). The SPIN uses a 5-point Likert scale with a higher score indicating greater social anxiety.

The second co-primary outcome, the HUSSAS, assesses social anxiety that is specific to the university context. We devised the HUSSAS specifically for the UniVRse project – the psychometric properties of this scale are being established as part of a separate study. The HUSSAS is inspired by the Liebowitz Social Anxiety Scale (LSAS) [43, 44], in which respondents rate their fear and avoidance of different social situations. The HUSSAS has 38 items describing different university-based situations (although this number may reduce following psychometric evaluation), each with three different rating scales: (a) fear: how anxious they feel in relation to the situation; (b) avoidance: how often they avoid the situation; and (c) desired avoidance: how often they would like to avoid the situation. The inclusion of the ‘desired avoidance’ subscale is to reflect that there may be some situations that participants are unable to avoid (i.e. compulsory parts of their studies) but wish they could. The questionnaire uses a 4-point Likert scale with a higher score indicating great fear or avoidance. A paper reporting on the psychometric properties of the HUSSAS is in preparation.

#### Secondary outcomes

We are also piloting the following secondary outcomes, which have been selected because they are hypothesised to also be targeted as part of the UniVRse intervention:

1. The Patient Health Questionnaire 9 (PHQ-9) [45] measures symptoms of depression. The PHQ-9 has 9 items using a 4-point Likert scale and evidenced strong internal consistency (Cronbach’s *α* = 0.89). A higher score indicates more depressive symptoms.
2. The Generalised Anxiety Disorder 7 (GAD-7) [46] measures symptoms of generalised anxiety. The GAD-7 has 7 items using a 4-point Likert scale, and past research shows very strong internal consistency (Cronbach’s *α* = 0.92). A higher score indicates more symptoms of generalised anxiety.
3. The Rathus Assertiveness Schedule (RAS) [47] measures general assertiveness. The RAS has 30 items with a 6-point Likert scale and established strong split-half reliability (*r* = 0.77; *p* < 0.01) [48]. The overall score may be positive or negative, with a higher positive score indicating greater assertiveness.
4. The Spreitzer’s Psychological Empowerment Scale competence subscale (SPES-c) [49] measures participants’ belief in their abilities in a specific context. The SPES-c was originally developed in relation to the workplace but has been adapted to refer to university studies for the present trial. The SPES-c subscale has three items with a 7-point Likert scale and strong internal consistency as found in previous studies (Cronbach’s *α* ≥ 0.81). A higher score indicates greater perceived competence.
5. The Short Instrument for measuring students’ Confidence with Key Skills (SICKS) [50] measures students’ confidence in their educational skills. The SICKS has 18 items with a 5-point Likert scale, divided into six subscales: (1) collaboration, (2) communication, (3) creativity, (4) self-direction, (5) critical thinking, and (6) technology for learning. All of the subscales have been shown to have strong internal consistency (Cronbach’s *α*s ≥ 0.80), with a higher score indicating greater confidence.
6. The Rosenberg Self-Esteem Scale (RSES) [51] measures self-esteem. The RSES has 10 items with a 4-point Likert scale, and well-established strong internal consistency (Cronbach’s *α*s ≥ 0.77) [52]. A higher score indicates greater self-esteem.
7. We will measure behavioural avoidance using students’ attendance to timetabled activities (e.g. lectures and seminars) collected via the ‘tap in’ system used at the trial site university. Students are required to log their attendance at all timetabled sessions using their student identification card. We will measure attendance data for the 2-week term time period preceding the T0 assessment, and the 2-week term time period after the T1 assessment.

#### Putative mechanisms

We will pilot the following constructs that have been selected as potential explanatory mechanisms for the hypothesised outcomes of the trial intervention:

1. The Question of Belonging (QoB) [53] measures the extent to which students feel a part of their university community. The QoB has three items with a 7-point Likert scale, and strong face validity—we will assess the psychometric properties within the present trial. A higher score indicates a greater sense of belonging.
2. The Identification with University (IwU) [54] measures the extent to which students perceive being a university student is consistent with their social identity. The IwU has four items with a 7-point Likert scale, and strong face validity—we will assess the psychometric properties within the present trial. A higher score indicates a greater perception that being a university student is consistent with their social identity.
3. The Domain-Specific Hope Scale social hope (DSHS-sh) and academic hope (DSHS-ah) subscales [55] measure self-agency and pathways thinking in relation to goals pertinent to (respectively) participants’ social relationships and academic study. Both subscales comprise 8 items with an 8-point Likert scale, and both achieve strong internal consistency (Cronbach’s *α* = 0.90) [55]. A higher score indicates greater hope in the respective domain.

#### Participant experience

As part of our aims to assess the acceptability of the UniVRse intervention, we are collecting data on participants’ experience of the intervention and using VR—specifically assessing for both positive experiences and side effects. These outcome measures will only be completed by those participants allocated to the immediate intervention arm:

1. The Increasing Access to Psychological Therapies Patient Experience Questionnaire (IAPT-PEQ) [56] measures participants’ perception of the intervention they received. The IAPT-PEQ has 8 items using a 5-point Likert scale, each of which is treated as a separate scale. A higher score indicates a more positive intervention experience.
2. The Edinburgh Adverse Effects of Psychological Therapy (EDAPT) [57] measures participants’ experience of side effects after receiving psychological therapy. Of the 42 potential EDAPT items, we have selected 31 items as potentially relevant to this project. Participants indicate whether the side effect applies to them or not (yes or no), and, where yes, rate the severity of the side effect using a 5-point Likert scale. Each item is reported individually with a higher score indicating greater severity of the side effect.
3. The Simulator Sickness Questionnaire (SSQ) [58] measures participants’ experience of side effects after using VR. The SSQ has 16 items with a 4-point Likert scale. Due to concerns regarding the conceptual overlap between the side effects of VR and the symptoms of anxiety, which may lead to measurement error, we will use the revised scoring and factor structure proposed by Bouchard et al. [59], which has demonstrated strong internal consistency (Cronbach’s *α* = 0.87).
4. The Friends and Family Test (FFT) [60] is a single-item measure of patient satisfaction that uses a yes/no response scale. Participants indicate whether they would recommend the intervention to friends and family experiencing a similar difficulty.

In addition to self-report measures of participant experience, we will obtain data from the VR kits to further inform our understanding of participants’ experience of the intervention. We will record the number of sessions completed, the virtual scenarios selected, levels completed within these scenarios, and time spent in VR. We will use these data to report rates of intervention completion, exposure, and dosage, as well as create an overall picture of how participants are using the UniVRse programme.

#### Cortisol

We will be inviting a sub-sample of 15 participants from each arm (30 in total) to provide saliva samples at T0 and T1 to measure levels of the stress hormone cortisol, and any changes in this between timepoints. Participants will be asked to collect their saliva samples, in their domestic setting, using salivettes. They will be asked to provide five samples per day for three consecutive days at T0 and again at T1 – a total of 30 saliva samples across their participation. These samples will need to be provided upon waking, and then 15-, 30-, 45-, and 60-min post awakening. In line with best practice guidance, to monitor sampling accuracy participants will also be asked to wear an activity-monitoring device to bed prior to the three collection days so we can verify the participants’ sleep and wake times [61]. The collection, storage, analysis, and disposal of the saliva samples will adhere to the Human Tissue Act (HTA).

#### Exit interviews

Participants who are randomised to receive the UniVRse intervention will be invited to complete an exit interview to be completed after the T1 assessment. The interview discussion guide combines the Change Interview protocol [62] and trial experience protocols [63] to explore participants’ experience of both the intervention and research procedures. We will invite participants in the intervention arm sequentially to complete an interview, while also trying to ensure this sub-sample is diverse in relation to key participant characteristics. We will cease recruitment when we have reached the recruitment target of 20, meeting sample size recommendations for a larger qualitative study [64].

### Data collection and storage

We will utilise a mixed-methods approach to address the project aims. We will collect the quantitative data at baseline (T0) and at a follow-up timepoint of 6 weeks post-randomisation (T1), and qualitative data after the post-randomisation assessment is complete (T2). The quantitative and qualitative data will be combined to understand the acceptability and participant experience of the UniVRse programme and trial.

Participants can choose to complete the assessments either in-person or online and with or without the support of a researcher. The flexibility in our data collection methods is based on patient and public involvement (PPI) feedback. Prior to the T0 assessment, participants will be presented with the participant information sheet (PIS) and asked to complete an eligibility assessment. The eligibility assessment will check whether participants meet the inclusion/exclusion criteria only. Where participants are eligible and interested, they will then be asked to provide written consent before completing the T0 assessment.

Upon completion of the T0 assessment, participants will be randomised to one of the trial arms. Participants will be notified of the outcome of the randomisation via email. The PI will liaise with the participant to arrange access to the UniVRse intervention. After the quantitative data collection is complete, participants in the intervention arm will be invited to complete an exit interview.

Any data that are collected as a hard copy will be scanned at the earliest opportunity and stored electronically on a password-protected university server, and the hard copy will be destroyed. Electronic assessment data will be collected using Qualtrics and then stored electronically on password-protected university computers. Participants will be assigned a unique participant identifier that will be used in place of their name on all assessments. Where data were collected using hard copies, they will be entered into Qualtrics by a member of the research team. We will check the quality of these data by reviewing the concordance between the data entered and the original file for a random sample of 20% of these assessments. Where we find errors in the data entry, we will review the correspondence between the source documents and data entered for the remaining assessments. We will correct any errors identified.

Cortisol concentrations will be determined by enzyme-linked immunosorbent assay (Salimetrics LLC, State College, PA, USA) at the Psychophysiology and Stress Research Group’s laboratory, University of Westminster. Saliva samples will be destroyed following assay.

The exit interviews will be audio-recorded and transcribed at the earliest opportunity. We will remove any identifying information at the point of transcription and use pseudonyms where necessary. Once the transcripts have been checked for accuracy, the original interview recording will be destroyed. The PI will act as the data custodian and oversee all aspects of data collection, management, and storage. All data procedures will adhere to the General Data Protection Regulation (GDPR) [65].

### Planned analysis

We will report the findings of this pilot trial in line with the appropriate Consolidated Standards of Reporting Trials (CONSORT) and pilot and feasibility extension [66, 67] guidelines. We will assess recruitment and retention rates by reporting the frequencies and percentages of participants that expressed an interest in the pilot trial (with reasons for disinterest where available), were deemed eligible or ineligible (with reasons for ineligibility), consented, and completed T0 and T1 assessments and the exit interviews. As part of this, we will record the frequencies and percentages of missing data for each outcome measure at each timepoint. Where available, we will collect information on the reasons for participants dropping out. Similarly, we will report on engagement with the UniVRse intervention and adherence to the intervention protocol using frequencies and percentages. We will report on the number and duration of sessions completed, and the number and typology of the scenarios and levels within each of these that the participants completed. This information will be recorded on the VR kits and downloaded by the research team.

In line with recommendations for pilot RCTs [68–70], we will not test any experimental hypotheses comparing the trial arms. We will summarise the primary and secondary outcomes, and putative mechanisms, at each timepoint using descriptive statistics and an intention-to-treat approach. For all outcomes, we will calculate the between-group Cohen’s *d* effect sizes with 95% confidence intervals, comparing scores at T1 for those in the intervention versus control arms, controlling for T0 scores using an ANCOVA. The between-group Cohen’s *d* effect sizes on the co-primary outcome will be used to establish whether there is a signal of efficacy in favour of the UniVRse intervention.

Saliva sampling accuracy will be determined by calculating the difference between electronically verified awakening and sampling times. Mixed regression modelling will be used to examine cortisol patterns between participants and within subjects. The quantitative participant experience data will be reported as frequencies and percentages. The qualitative data will be analysed using Thematic Analysis, following the Braun and Clarke protocols [71]. This six-stage approach to analysis involves immersion with the data, coding of the smallest units of meaning within each transcript, and the clustering of codes into themes and sub-themes within and across transcripts. The final thematic structure will be reported alongside illustrative quotes from participants. All participants will be asked to provide consent for their direct quotes to be used within any reports that come from this project. The qualitative data will allow us to explore acceptability and provide insights on if and how we can refine the research and intervention protocols if we were to progress to a definitive trial.

A definitive trial will be justified if the following criteria are satisfied: (1) we collect complete data on a co-primary outcome for at least 80% of participants; (2) attrition rates across the trial do not exceed 40%; and (3) the between-group effect size on the co-primary outcomes favours the UniVRse intervention over the wait-list control condition.

### Public and patient involvement

The current pilot trial is the second phase in the wider UniVRse project. The first phase was the co-production of the UniVRse intervention with a group of six students enrolled at the trial site, who identified as lacking confidence and/or experiencing social anxiety at university. The students responded to a university-wide advert asking for consultants on the UniVRse project. The final group was selected based on their lived experience and to represent a diversity of sociodemographic backgrounds. All of the students were paid for their time spent attending meetings and for any additional preparatory work. The meetings were conducted both in person and online. At least one member of the research team and a representative from the VR design company were present at every meeting.

The two key questions that the students were asked to consult on were (1) what aspects of university life were the most anxiety-inducing and (2) what are the anxiety-related triggers associated with these university-based situations? When discussing these questions, the students reflected that making these decisions based on anxiety alone would not necessarily make for a meaningful intervention. Instead, they selected the scenarios for the UniVRse intervention based on those that made them feel anxious but that they also cared about and in which they wanted to feel more confident. For example, being in the campus foyer can be anxiety-inducing, but the students prioritised being in a group-working scenario, for the latter has greater implications for other areas of their life and is an important skill to develop for future employability. The outcome of our discussions was the UniVRse intervention storyboard, outlining the four university-based scenarios and details of the five graded levels within these.

The students also contributed to the trial design, including recruitment and data collection methods. There are several specific examples where their feedback was implemented. The students reflected that social anxiety manifests in different ways—for some it may mean that the idea of meeting the research team to complete assessments could be anxiety-inducing, whereas for others they would want to complete the assessments with the support of a member of the research team to check they have understood the assessments. We have therefore developed a protocol whereby students can choose to complete assessments in-person or online and with or without the support of a member of the research team. Similarly, they shared that there is still a stigma around poor mental health and that can prevent students from seeking support—including participating in the UniVRse project. We therefore agreed to remove any unnecessary clinical language from participant-facing materials, i.e. describing UniVRse as a programme to help students feel ‘more confident’ instead of feeling ‘less anxious’.

Moving forward, students will continue to provide oversight of the UniVRse trial. We have recruited two students to sit on the Trial Steering Committee and provide their lived experience perspectives. These students will be paid for their time. We made the decision to recruit different students from those involved in phase 1 of the project, considering that their involvement in the coproduction may present a conflict of interest.

### Research governance

The pilot trial protocol has been produced in line with the Standard Protocol Items: Recommendations for Interventional Trials (SPIRIT) [72, 73] and CONSORT [66, 67] guidelines. The UniVRse project is funded by the Office for Students (Reference: DMH05) and has received ethical approval from the University of Westminster College Research Ethics Committee (C-REC; reference: ETH2122-3503).

We have established a Trial Steering Committee (TSC) to oversee the UniVRse pilot trial. The TSC has been set up in line with guidance from the Medical Research Council [74]. The TSC is led by an independent chair, and includes representatives with expertise in trial management, student mental health, VR, as well as a statistician, Clinical Psychologist, and two students. The Clinical Psychologist on the TSC will act as the independent clinician who will review any adverse events that occur during the trial. Although there are no specific guidelines for the reporting of adverse events in digital mental health trials, we will adhere to Good Clinical Practice guidelines [74] and follow the recommendations of a recent scoping review in this area [75]. We will assess and rate each adverse event internally in terms of the seriousness and relatedness to the intervention and/or study procedures, and then share these reports with the independent clinician to ratify or challenge any decisions. We will also capture further information on adverse events and experiences using the SSQ and EDAPT (see Section 7.6.2). We will record and report all adverse events in the final report of the trial results, irrespective of their seriousness and/or relatedness.

### Dissemination

We will write up the results of the pilot trial for publication in an academic journal and present the findings at relevant academic conferences. We are also committed to sharing our findings beyond the academic community by producing alternative reports of the results, including blog posts and project summaries. We will share these reports via third sector and student organisations, the UniVRse website (https://blog.westminster.ac.uk/univrse/), and social media channels. As part of our dissemination activities, we also plan to hold in-person and virtual seminars where we present the findings of the trial and showcase the UniVRse intervention.

## Discussion

The results of this pilot RCT will determine whether the research and intervention protocols are acceptable to participants, establish whether a definitive trial is justified, and identify the parameters needed for the sample size calculations for a definitive trial. If the a priori defined criteria for progression to a definitive trial are met, then we will refine the research protocols and intervention in light of the findings of the pilot trial as needed. We will then pursue funding to conduct a definitive trial of the UniVRse intervention.

If we find the UniVRse intervention to be an effective intervention for reducing global and context-specific social anxiety amongst students within a definitive trial, then we will seek to implement the intervention across UK universities. The self-help nature of the UniVRse intervention means it does not require input from a practitioner to deliver it. In addition, VR kits are becoming more affordable and commonplace within universities and domestic settings. We have committed to offering all UK universities a year-long license for the UniVRse intervention for free. The UniVRse intervention has the potential to improve the mental health of students and their experience and engagement with all aspects of university life.

### Trial status

The trial opened for recruitment in January 2024.

## Data Availability

Data sharing is not applicable to this article as no datasets were generated or analysed during the current study.

## Abbreviations

CBT: Cognitive Behaviour Therapy
DSHS-ah: Domain Specific Hope Scale academic hope subscale
DSHS-sh: Domain Specific Hope Scale social hope subscale
EDAPT: EDinburgh Adverse effects of Psychological Therapy
FFT: Friends and Family Test
GAD7: Generalised Anxiety Disorder 7
HTA: Human Tissue Act
HUSSAS: Hazell University-Specific Social Anxiety Scale
IAPT: Increasing Access to Psychological Therapies
IwU: Identification with University
PEQ: Patient Experience Questionnaire
PHQ9: Patient Health Questionnaire 9
PPI: Patient and public involvement
PI: Principal investigator
QoB: Question of Belonging
RAS: Rathus Assertiveness Schedule
RCT: Randomised controlled trial
RSES: Rosenberg Self-Esteem Scale
SICKS: Short Instrument for measuring students’ Confidence with Key Skills
SPES-c: Spreitzer’s Psychological Empowerment Scale competence subscale
SPIN: Social Phobia Inventory
SSQ: Simulator Sickness Questionnaire
TSC: Trial Steering Committee
VR: Virtual reality
